# A Case of Enteric Fever

**Published:** 1908-12-26

**Authors:** 


### The General Practitioner's Column.

[Contributions to this Column are invited, and if accepted will be paid for.]

### A CASE OF ENTERIC FEVER.

As there is no disease that presents itself in more varieties of form than enteric fever, records of abnormal cases are generally instructive.

H. W., æt. 28, was surgeon on a Cape Mail steamer which left Southampton on July 3, 190—. About a week later he began to feel "out of sorts," but had no definite symptom of illness until July 17, when towards evening he had a slight shivering attack, and found his temperature to be 101°. For the next two days his temperature was 101-2°, the only other symptoms being constipation and headache. He did not keep to bed, and was able to see patients. On the 20th the ship arrived at Cape Town. His temperature that morning was 102°, but he went about the ship attending to the duties incidental to arrival in port, and was present at a dinner party in the evening. That night the temperature was 103°, and recognising now that his illness was something more than a "feverish cold," he kept to his berth, and was seen by one or two doctors. For three days the symptoms continued, fever, constipation, headache, but nothing more. On the 24th, about 4 a.m., he awoke in a profuse sweat, and found his temperature 98°. As the ship was now leaving to go round the coast he was advised to remain behind in Cape Town, but feeling better he decided to go on. There was no further rise of temperature, but for two nights he had a profuse outbreak of urticaria. On the 26th he resumed his duties as ship's surgeon. For two or three weeks nothing further happened; he felt unaccountably weak at times, but the temperature remained normal, with one exception, when it rose to 100° after a walk on shore at Durban. On August 11 the ship left Cape Town homeward bound. On the 13th he played at "deck-cricket" for a short time, and his temperature rose at night to 102°. Next day he remained in bed, but his temperature reached 103°. Fortunately a medical man was on board as a passenger, and he undertook charge of the sick surgeon and also of the ship. During the next week the patient's temperature ranged between 101° in morning and 103° at night. Other symptoms were constipation, headache, and insomnia, but there was neither abdominal pain nor tenderness, and no spots were seen. The spleen was not felt. But a diagnosis of enteric was agreed upon, and the case treated accordingly. From August 20 to 26 the temperature decreased and one or two "pea-soup" stools were passed. Southampton was reached on August 27, and the patient was transferred to a nursing home, where he remained for four weeks and made an uneventful recovery.

*Remarks.*—That the case was one of mild enteric fever from the beginning there can be little doubt. The initial attack, after lasting six days, "aborted," and the outbreak of urticaria which immediately followed was due presumably to disturbance of the alimentary functions. The patient did not realise then the nature of his attack, and took no after-precautions in the way of rest and diet. In consequence he had a relapse eighteen days later, which lasted longer and proved more severe than the initial attack.

A sequel of some interest remains to be told. Some four years after his illness H. W. held a demonstratorship in a bacteriological laboratory, and then found that his blood in a dilution of 1 in 20 caused agglutination of the laboratory strains of typhoid bacilli in about fifteen minutes, and he was able at any time to show the Widal reaction to students when the serum of an enteric case was not obtainable. This agglutination power gradually weakened, and two years later had altogether disappeared. That it should have persisted so long after the attack of enteric was certainly unusual, but as the reaction was obtainable with three different strains of typhoid bacilli (one of which was isolated by himself from the spleen of a fatal case of enteric) there could be no doubt as to its validity.

